# Heterogeneity of Necrotic Changes between Cortical and Cancellous Bone in Mandibular Osteoradionecrosis: A Histopathological Analysis of Resection Margin after Segmental Mandibulectomy

**DOI:** 10.1155/2017/3125842

**Published:** 2017-08-30

**Authors:** Masaya Akashi, Kazunobu Hashikawa, Satoshi Wanifuchi, Junya Kusumoto, Manabu Shigeoka, Shungo Furudoi, Hiroto Terashi, Takahide Komori

**Affiliations:** ^1^Department of Oral and Maxillofacial Surgery, Kobe University Graduate School of Medicine, Kobe, Japan; ^2^Department of Plastic Surgery, Kobe University Graduate School of Medicine, Kobe, Japan; ^3^Division of Pathology, Kobe University Graduate School of Medicine, Kobe, Japan

## Abstract

**Background:**

This study aimed to analyze differences in necrotic changes between cortical and cancellous bone in resection margins after segmental mandibulectomy for advanced mandibular osteoradionecrosis.

**Methods:**

Anteroposterior bone specimens from eleven patients who underwent segmental mandibulectomy with simultaneous free fibula flap reconstruction for advanced osteoradionecrosis were analyzed histopathologically for the presence of necrotic bone based on the presence of blood vessels within Haversian canals.

**Results:**

Ten of eleven (91%) cortices near the inferior border of the mandible at the anterior margins were necrotic. All cancellous bones at the anterior margins were viable. Seven of eleven (64%) cortices near the inferior border of the mandible at the posterior margins were necrotic. Three of eleven (27%) cancellous bones at the posterior margins were necrotic.

**Conclusion:**

Necrotic changes are more prevalent in cortices than in cancellous bones in mandibular osteoradionecrosis, probably due to a decrease of periosteal blood supply caused by radiotherapy.

## 1. Introduction

Radiotherapy (RT) has played an indispensable role in the modern treatment of head and neck malignancies. Among the most devastating complications of RT is osteoradionecrosis (ORN) of the jaw. The most recent large cohort study reported the prevalence of ORN to be 6.2% in patients who underwent RT for oral cancer [1]. ORN can predispose patients to recurrent infection, orocutaneous fistula, pathological fracture, and injury to inferior alveolar nerve (IAN). Patients with these complications experience several symptoms including trismus, severe pain, chronic drainage, disfigurement, and insufficient nutrition, resulting in serious impairment in quality of life [2, 3].

Several classifications have been proposed in literature to facilitate the diagnosis of ORN [2–10]. The only reliable treatment option for advanced ORN (resorption of the inferior border of the mandible, orocutaneous fistula, and pathological fracture) is surgical debridement and free flap reconstruction [3, 11–13]. To achieve a successful outcome for this surgery, both adequate bone resection and survival of the transferred flap are essential. The extent of resection is generally determined by presence of bleeding at resected edges; however, ORN can recur at viable margins [14]. To understand the underlying pathological mechanisms of ORN, a histopathological study is needed.

Previous studies performed histological analysis of ORN [15–18]; however the bone specimens analyzed were obtained during sequestrectomy or decortication. These studies lacked detailed information regarding location of obtained bone specimens. In the only histological study analyzing the resection margins after segmental mandibulectomy by Zaghi et al. [14], the anteroposterior location of the bone specimens and differences between cortical and cancellous bone were not described.

This histopathological study analyzed the anteroposterior margins after segmental mandibulectomy for advanced ORN. The purpose of this study was to evaluate the necrotic changes in cortical and cancellous bones at resection margins and proximal areas of bone destruction.

## 2. Materials and Methods

We enrolled eleven consecutive patients who underwent segmental mandibulectomy for surgical debridement and simultaneous free fibula osteocutaneous flap transfer for treatment of advanced mandibular ORN in our department between July 2013 and August 2016. No patients who underwent surgical intervention for ORN were excluded. ORN was defined as a nonhealing exposure of bone of at least 6 months' duration [19]. All patients had various symptoms, including pain, infection, trismus, and difficulty eating, and were resistant to conservative therapy. Severe pain (e.g., lightning pain causing sleep deprivation) caused by damage to the IAN was regarded as the most important finding guiding the decision to intervene surgically. The extent of mandibulectomy with adequate safety margins (more than 10 mm) from apparent osteolytic areas was determined by preoperative thin-slice computed tomographic imaging. The Medical Ethics Committee of Kobe University Hospital approved this study. All subjects gave written informed consent to release clinical information and bone samples for the study.

The following epidemiological data were gathered retrospectively from medical charts: age, sex, pathological diagnosis, primary tumor sites, types of RT, radiation dose, chemotherapy, surgery for primary tumor, time interval between the end of RT and surgery for ORN, the existence of pathological fracture, location of ORN, and surgical findings (the extent of segmental mandibulectomy and bleeding at the resection margins). Segmental mandibulectomy defects were classified according to the “CAT classification” used in our previous reports [20, 21]. Briefly, defects were classified on the basis of three anatomical landmarks: the mental tubercle [T], mandibular angle [A], and condyle [C]. The lesions that did not include tubercle, angle, and condyle were classified as mandibular “body.”

## 3. Histopathological Analysis

All bone specimens were decalcified and fixed in formalin but not frozen. The details of decalcification method were as follows: formic acid (98%) (Wako, Osaka, Japan) was diluted to 10% by distilled water. The bone specimens were immersed in 10% formic acid with ion exchange resin and treated by ultrasonic histoprocessor Histra-DC (Jokoh, Tokyo, Japan) for several days to several weeks. Thin sections were obtained from paraffin blocks and stained with hematoxylin and eosin for light microscopy.

**Figure 1:**
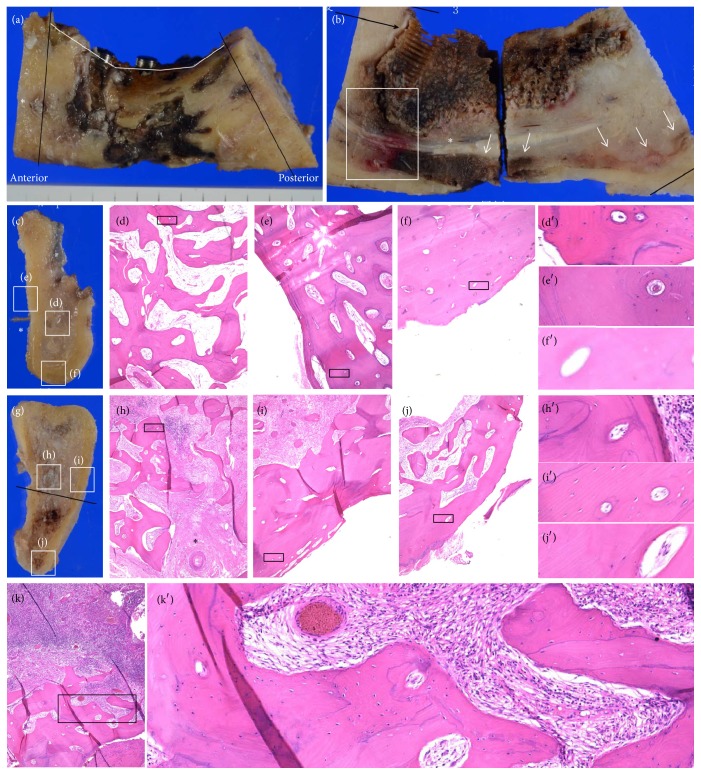
Clinical and histopathological images of a representative case (patient number 10). (a) A resected bone specimen. The anterior and posterior specimens were prepared apart from the true resection margin to avoid heat artifacts caused by the surgical saw (black lines). Mental foramen (*∗*). (b) A sagittal section. The most advanced area of bone destruction (white box). Mandibular canal (*∗*). Color change was found along the cortical bone of the inferior border of the mandible (arrows). (c) Anterior margin. Mental nerve (*∗*). (d) Viable cancellous bone in anterior margin. (e) Viable cortical bone at the middle level of the mandible at the anterior margin. (f) Necrotic cortical bone near the inferior border of the mandible. (d′–f′) Enlarged views. Viable bone evident with blood vessels within Haversian canals (d′ and e′) and necrotic bone evident with empty Haversian canal (f′). (g) Posterior margin. (h) Cancellous bone, (i) cortical bone at the middle level of the mandible, and (j) cortical bone near the inferior border of the mandible. (h′–j′) Enlarged views showing viable bone evident with blood vessels within Haversian canals. (k) Cancellous bone near the most advanced area of bone destruction shown in white box in (b). (k′) Enlarged view showing viable bone evident with osteocyte nuclei within lacunae. All specimens were stained with hematoxylin and eosin, original magnification ×4.

To analyze the differences in necrotic changes among the anteroposterior locations, thin sections from the anterior and posterior margins of segmental mandibulectomies were used for observations, as shown in Figure 1. The cross sections of the anterior and posterior margins were prepared separately from the true resection margins to avoid artifact caused by the heat of the surgical saw (Figure 1(a)). To analyze the differences between cortical and cancellous bone, we examined cancellous bone within the bone marrow cavity at the middle level of the mandible (Figures 1(c) and 1(g)) in each section. To analyze the differences of cortices among the craniocaudal locations, we examined cortical bone at the middle level of the mandible and near the inferior border of the mandible in each thin section at the anterior and posterior margins (Figures 1(c) and 1(g)). To analyze viability near the center of lesion, we examined cancellous bone near the most advanced area of bone destruction (Figures 1(b) and 1(k)).

**Figure 2:**
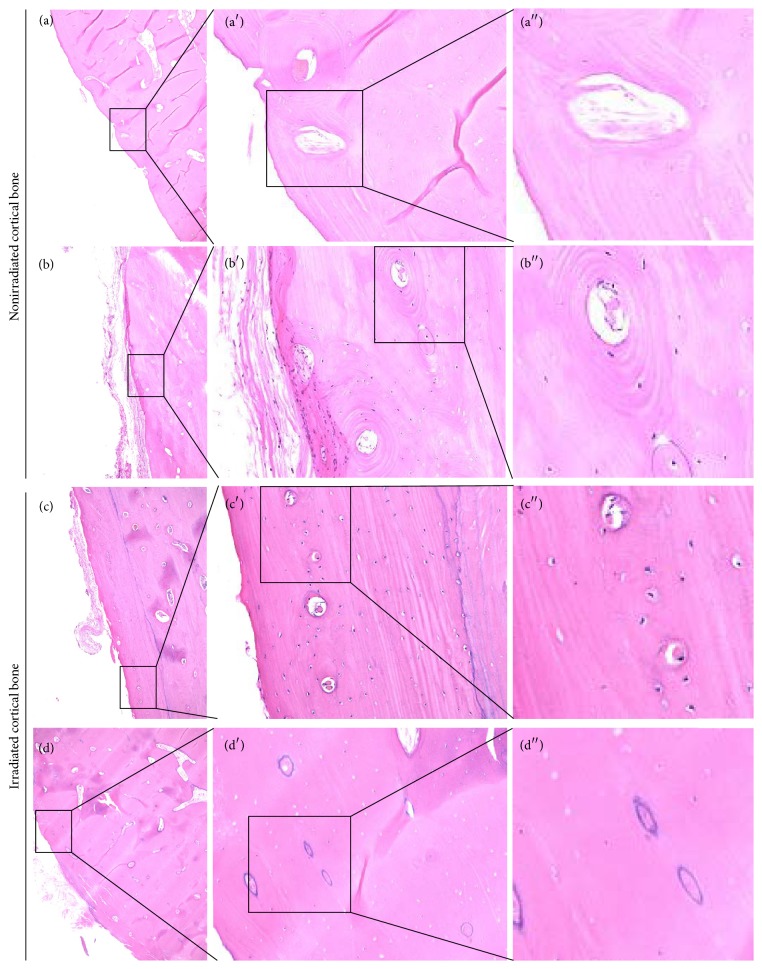
Empty lacunae were frequently found in normal viable cortical bone. (a) Nonirradiated cortical bone specimen. Lack of osteocyte nuclei was found despite presence of viable blood vessels in Haversian canals. (b) Nonirradiated viable cortical bone evident with osteocyte nuclei within lacunae and blood vessels in Haversian canals. (c and d) Irradiated cortical bone obtained from a patient who underwent segmental mandibulectomy for advanced ORN. (c) Viable cortical bone evident with osteocyte nuclei within lacunae and blood vessels in Haversian canals. (d) Necrotic bone evident with no viable blood vessels within Haversian canals.

A previous study analyzed bone viability based on microscopic findings of osteocyte nuclei within lacunae and presence of viable blood vessels within Haversian canals [14]. To confirm the validity of differentiation between viable and necrotic cortical bone, we performed a preliminary microscopic analysis of randomly selected non-irradiated bone specimens obtained from patients who underwent segmental mandibulectomy for benign tumors or oral malignancies, but did not receive RT. As shown in Figure 2(a), empty lacunae were frequently found in non-irradiated viable cortical bone with obvious viable blood vessels in Haversian canals. Osteocyte number varies with age [22]. Therefore, this study focused on the presence of blood vessels and red blood cells within Haversian canals rather than the number of osteocyte nuclei within lacunae. Cortical bone specimens with complete obstruction of Haversian canals as shown in Figure 2(d) were regarded as “necrotic”. Conversely, the viability of cancellous bone within bone marrow cavity was determined by the presence of osteocyte nuclei within lacunae. Image acquisition of whole bone specimens (×4) was performed with a BZ-X 700 (Keyence, Osaka, Japan). Analysis of necrotic changes was independently performed by four observers (MA, SW, JK, and MS). MA and JK are oral and maxillofacial surgeons with more than ten years of experience, SW is a graduate fellow in our department, and MS is an oral pathologist with more than ten years of experience. There were discrepant results among the four observers in some specimens, as detailed below. In specimens with discrepant results, a mixture of viable and necrotic bone was found. Those specimens were defined as “heterogeneously necrotic” in this study.

## 4. Results

Clinical characteristics of patients in this study are shown in Table 1. Eleven consecutive patients underwent surgical resection and simultaneous reconstruction with free fibula osteocutaneous flap for advanced mandibular ORN. Ten were male, and median age of the eleven patients was 65 years (range, 58–80 years). The median radiation dose was 66 Gy (range, 60–81 Gy). The median time interval between the end of RT and surgery for ORN was 84 months (range, 6–152 months). One patient (number 5) required surgical intervention after having developed a cutaneous fistula and subsequent mandibular pathological fracture shortly after completion of intensity-modulated radiotherapy. The location of ORN was ipsilateral to the primary tumor in four patients (36%), and contralateral in seven (64%). In operative findings, bleeding after segmental mandibulectomy was noted. There was one total flap loss in patient number 4.

The remaining ten flaps survived.

Table 2 shows the histopathological results of bone samples. The concordance rate of the differentiation of viable and necrotic bone among four observers was 73–100%. Cancellous bones at the anterior margins were viable in all specimens. In contrast, cortical bone at the middle level of the mandible of the anterior margin was viable in only four of eleven specimens (36%). Cortical bone near the inferior border of the mandible at the anterior margin was “necrotic” or “heterogeneously necrotic” in ten of eleven specimens (91%). Cancellous bones at the posterior margins were viable in eight of eleven specimens (73%). Evidence of viability was observed in five of eleven specimens in cortical bones at the middle level (45%) and four of eleven specimens near the inferior border of the mandible (36%) at the posterior margin. Cancellous bone near the most advanced area of bone destruction was viable in seven of eleven specimens (64%).

Representative clinical and histopathological images are shown in Figure 1. Figures 1(a) and 1(b) show a bone sample from patient number 10 who received concomitant chemoradiotherapy for oropharyngeal carcinoma 7 years prior to surgery. Bone destruction around osseointegrated dental implants extended to the inferior border of the mandible. Color change due to necrosis was found along the cortical bone of the inferior border of the mandible as well as in alveolar bone (Figure 1(b)). Specimens of cancellous bone at the anterior (Figure 1(d)) and posterior (Figure 1(h)) margins were viable. Although cortical bone was viable at the middle level of the mandible at the anterior (Figure 1(e)) and posterior (Figure 1(i)) margins, as well as near the inferior border of the mandible of the posterior margin (Figure 1(j)), cortical bone near the inferior border of the mandible was necrotic (Figure 1(f)). Cancellous bone near the most advanced area of bone destruction was viable (Figure 1(k)).

**Figure 3:**
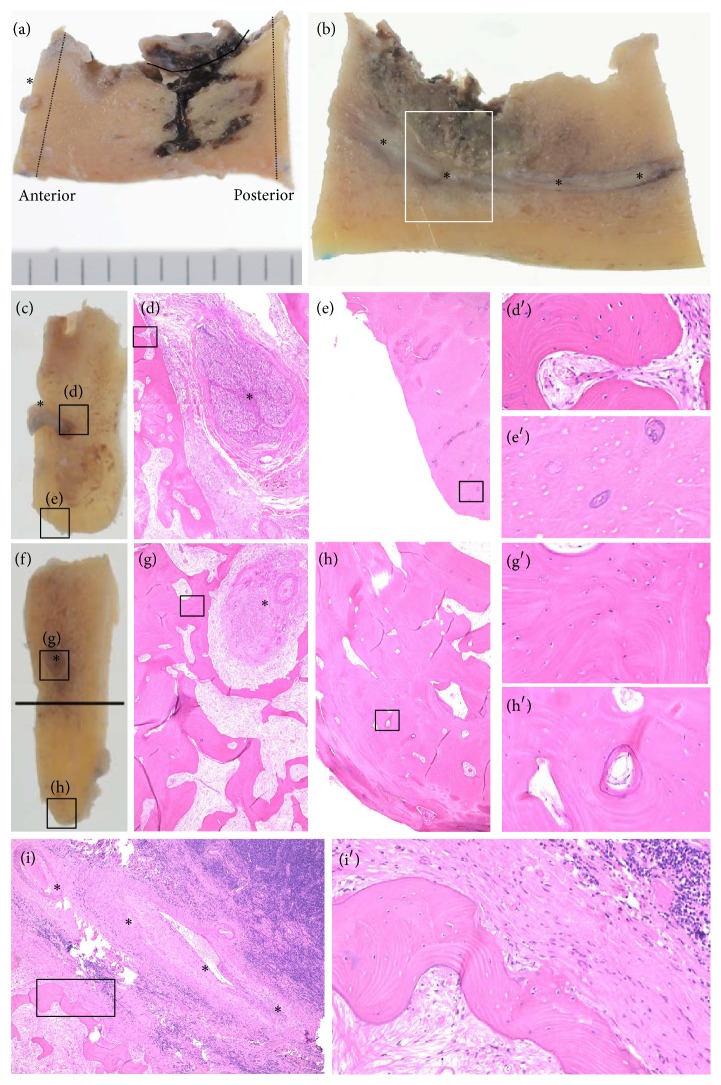
Clinical and histopathological images of a representative case (patient number 9). (a) A resected bone specimen. Mental foramen (*∗*). (b) A sagittal section. The most advanced area of bone destruction (white box). *∗* indicates mandibular canal. (c) Anterior margin. Mental nerve (*∗*). (d) Viable cancellous bone at the anterior margin. Mental nerve (*∗*). (e) Necrotic cortical bone near the inferior border of the mandible. (d′ and e′) Enlarged views. (f) Posterior margin. (g) Cancellous bone and (h) cortical bone near the inferior border of the mandible. Inferior alveolar nerve (*∗*). (g′) Enlarged view showing viable bone evident with osteocyte nuclei within lacunae. (h′) Enlarged view showing blood vessels within Haversian canals. However, a mixture of viable and necrotic bones was found. Therefore, the classification is “heterogeneously necrotic.” (i) Cancellous bone near the most advanced area of bone destruction shown in white box in (b). (i′) Enlarged view showing viable bone evident with osteocyte nuclei within lacunae. Inferior alveolar nerve (*∗*). All specimens were stained with hematoxylin and eosin, original magnification ×4.

Figure 3 shows bone specimens from patient number 9. Cancellous bone was viable surrounding the mental nerve at the anterior margin (Figure 3(d)) and the mandibular canal at the posterior margin (Figure 3(g)). However, cortical bone near the inferior border of the mandible at the anterior margin was necrotic (Figure 3(e)). Cortical bone near the inferior border of the mandible at the posterior margin was “heterogeneously necrotic” (Figure 3(h)). Bone near the osteolytic area was viable (Figure 3(i)).

**Figure 4:**
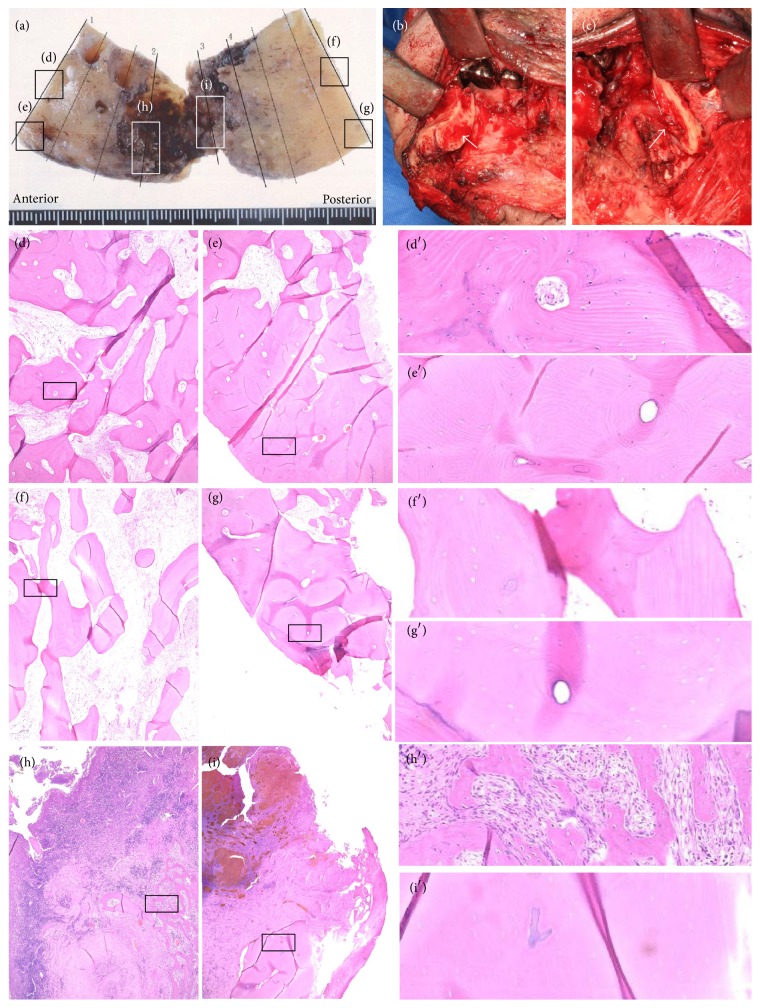
Clinical and histopathological images of patient number 8. (a) A resected bone specimen. (b and c) Surgical findings. Bleeding was found at the anterior (b) and posterior (c) margins. (d) The cancellous bone at the anterior margin was viable. Cortical bone at the anterior margin (e) and cancellous bone at the posterior margin (f) were necrotic. (g) Cortical bone at the posterior margin was “heterogeneously necrotic.” The viable bone was found near the most advanced area of bone destruction (h), whereas the bone in the center of osteolysis was necrotic (i). (d′–i′) Enlarged views. All specimens were stained with hematoxylin and eosin, original magnification ×4.

Figure 4 shows bone specimens from patient number 8. During surgery, bleeding from bone marrow both at anterior (Figure 4(b)) and posterior (Figure 4(c)) margins was identified. Histopathological examination revealed obvious hypocellular fibrosis within the bone marrow cavity (Figures 4(d) and 4(f)). Except for viable cancellous bone at the anterior margin (Figure 4(d)), bone samples of cortical bone at the anterior margin (Figure 4(e)) and cancellous bone at the posterior margin (Figure 4(g)) were necrotic. Cancellous bone near the inferior border of the mandible at the posterior margin (Figure 4(f)) was “heterogeneously necrotic”. Viable bone was observed near the most advanced area of bone destruction (Figure 4(h)), whereas bone in the center of osteolysis was necrotic (Figure 4(i)).

The median follow-up period was 18 months (range, 11–48 months). The progression of necrosis after surgery occurred at the posterior viable margin only in one patient (number 3), but ceased in a few months. In contrast, there were cases in which good bone union between fibula flaps and the resection margins diagnosed histologically as necrotic. Overall, the incidence of progression of bone resorption arising from resection margins after surgery was 9%.

## 5. Discussion

This study analyzed the anterior and posterior margins of segmental mandibulectomies and identified the differences in necrotic changes between cortical and cancellous bone in advanced mandibular ORN. To our knowledge, there have been no prior studies analyzing the resection margins at both ends, or analyses of the differences in necrotic changes between cortical and cancellous bone in advanced ORN. This study illustrates the heterogeneity of necrotic changes in advanced mandibular ORN. Necrotic change is more prevalent in cortical bone than in cancellous bone. We report a rate of 91% necrotic change in cortical bone at the inferior border of the anterior margin. By contrast, all cancellous bones at the anterior margins were viable. The percentage of viable cancellous bones near the most advanced area of bone destruction was unexpectedly high: 64%. We also note that necrotic changes of cancellous bone at the posterior margins were found in three bone specimens (27%).

The heterogeneity of bone viability between cortical and cancellous bone was probably due to differences in blood supply. The facial artery is the major extraosseous source of blood to mandibular body. Therefore, “swing” osteotomy, ligation of the facial artery, and RT may all affect blood flow to the mandible [23]. Considering the importance of blood supply to bone repair in the mandibular cortex, Saka et al. [24] performed a study of blood flow in human mandibles. They divided the mandible into three anatomical zones as follows: Zone I: mandibular body, beginning in the symphysis and ending at the connecting line between the retromolar area and the mandibular angle; Zone II: the caudal part of the mandibular ramus, located dorsally and cranially to Zone I, extending to the condylar base; and Zone III: the condyle (i.e., the condylar process with the mandibular head, located cranially to Zone II). The main blood supply to the cortices in Zone I is periosteal, deriving from the mental, submental, and sublingual arteries. Collateral supply is endosteal and periosteal, deriving from the inferior alveolar and mental arteries. In this study, almost all resected specimens were classified as Zone I. Necrotic changes, especially in the cortices near the inferior border of the mandible at the anterior margins, are probably due to reduction of periosteal blood flow caused by RT. We did not find a greater occurrence of necrotic changes of cortices in patients who underwent ipsilateral neck dissection for primary carcinomas, as compared with other patients. This suggests that necrotic changes of cortices are caused by damage to the microcirculation throughout the entire periosteum, rather than the absence of one dominant nutrient artery (i.e., the facial artery).

Poor outcome in ORN is associated with minimal surgical debridement alone [25]. The refractoriness of ORN to minimal debridement may be because necrotic changes occur mostly in cortical bone rather than in cancellous bone. We unexpectedly found that cancellous bone near the site of bone destruction was viable at a high frequency. This finding suggests that surgeons may find bleeding from the bone marrow in surgical margins of ORN during surgery, irrespective of surgical extent.

A review by Jacobson et al. [3] classified ORN into early, intermediate, and advanced stages. Early ORN should be managed conservatively with local wound care and the administration of antibiotics. Advanced ORN requires surgical management with wide extirpation of disease and simultaneous free flap reconstruction [3, 13]. Appropriate definitive treatment of intermediate stage ORN remains uncertain [3]. Jacobson et al. [3] recommend that, for intermediate ORN, all necrotic bone be debrided transorally up to the bleeding edge, and the wound should be primarily closed. We mostly agree with their recommendation; however the results of our study suggest that repeated minimal debridement in intermediate ORN creates the risk of advancing fragility of the residual bone. This is because necrotic changes are dominant in cortices near the inferior border of the mandible.

The “hypoxic-hypocellular-hypovascular” theory of ORN pathophysiology was proposed by Marx in 1983 [10]. In 1990, Bras et al. [26] analyzed sequestrectomy specimens and reported that the dominant feature of mandibular ORN was ischemic necrosis due to obliteration of the inferior alveolar artery by RT. Revascularization by branches of the facial artery was disturbed by vascular disease and periosteal damage caused by RT [26]. They also proposed that the most vulnerable part of the mandible was the buccal cortex of the premolar, molar, and retromolar regions [26].

Imbalance of bone remodeling is another pathogenic feature of ORN. Bone remodeling involves a balance between osteoclast resorption and osteoblast deposition [27]. Local external irradiation causes a decrease in numbers of osteocytes and osteoblasts [27]. A study using mini pigs by Xu et al. [27] suggested that the decrease of local blood flow due to microvessel damage may be an initiating factor for ORN, followed by secondary induction of an imbalance in bone remodeling.

Another model for pathogenesis of ORN is the “fibroatrophic” theory [28]. This theory proposes three clinical and histopathological phases: a prefibrotic specific inflammatory phase, a constitutive fibrotic cellular phase, and a matrix densification and remodeling phase, possibly ending in terminal tissue necrosis [28]. A histopathological study of human bone samples of ORN obtained from sequestrectomy samples suggested that ORN is characterized by increased collagen deposition (fibrosis) [17]. In our study, we found that the severity of fibrosis within the bone marrow cavity was different in each patient. Further analysis of fibrosis in advanced ORN is needed.

The determination of resection extent for ORN remains an unresolved issue of importance. One serious postoperative complication of ORN surgery is residual, or recurrent ORN. Suh et al. [29] found a 25% rate of recurrent ORN after segmental mandibulectomy. Zaghi et al. [14] found that the presence of residual necrotic bone at resection margins of segmental mandibulectomies did not correlate with the recurrence of ORN. This was because all recurrence of ORN occurred in viable resection margins, and there was no progression of ORN at the resection margins in residual nonviable bone [14]. Our study suggests that complete extirpation of necrotic bone is sometimes impossible, despite resection of the destroyed bone area with a wide safety margin (Figure 4). Recurrence of ORN may be related to factors (e.g., infection) other than the presence of residual necrotic bone at the resection margin. The limitation of this study is the relatively short period of follow-up (median 18 months). Therefore, our study could not evaluate the recurrence and bone union between the residual mandible and transferred fibula flap through long-term observation. In the future we hope to perform a long-term follow-up study looking at the rate of ORN recurrence and union of bone junctions.

## 6. Conclusion

In ORN cases requiring surgical intervention, cortical necrosis, especially near the inferior border of the mandible, was more common than necrosis of cancellous bone. Cortical necrosis was probably due to a decrease in periosteal blood supply. Cancellous bone, even near the area of bone destruction, was viable in some cases. In cases of severe fibrotic ORN, complete extirpation of necrotic bone may be difficult even though segmental mandibulectomy with wide safety margins is performed.

## Figures and Tables

**Table 1:** Clinical characteristics of patients.

Case	Sex	Age	Pathological diagnosis	Primary site	Types of RT/dose (Gy)	Chemotherapy	Surgery for primary tumor	Time interval (months)^*∗*^	Pathological fracture	Lesion location^*∗∗*^	Extent of SM^*∗∗∗*^
1	M	71	SCC	Oral cavity	Conventional/61.5	—	Tumor resection biND/RF	120	+	Ipsilateral	Body
2	M	58	SCC	Oropharynx	Conventional/70	CDDP	uniND	104	−	Contralateral	Body
3	M	70	SCC	Neck (unknown primary)	Conventional/66	CDDP	uniND	75	−	Contralateral	Body
4	M	62	SCC	Oropharynx	Conventional/70	CDDP/5-FU	—	78	+	Contralateral	A
5	F	80	SCC	Oral cavity	IMRT/60	—	Tumor resection uniND/RF	6	+	Ipsilateral	AT
6	M	66	AC	Neck (unknown primary)	Conventional/60	—	uniND	121	+	Contralateral	A
7	M	65	SCC	Oropharynx	Conventional/60	CDDP/NDP	Tumor resection uniND/RF	137	−	Ipsilateral	A
8	M	64	SCC	Neck (unknown primary)	Conventional/81	CDDP/5-FU	—	152	+	Ipsilateral	AT
9	M	63	SCC	Nasopharynx	Conventional/70	CDDP/5-FU	—	56	−	Contralateral	Body
10	M	63	SCC	Oropharynx	Conventional/70	CDDP	—	84	+	Contralateral	Body
11	M	74	SCC	Oropharynx	Conventional/66	CDDP/5-FU	Tumor resection biND/RAMC	71	−	Contralateral	Body

RT, radiotherapy; ORN, osteoradionecrosis; SCC, squamous cell carcinoma; AC, adenocarcinoma; IMRT, intensity-modulated radiotherapy; CDDP, cisplatin; 5-FU, fluorouracil; NDP, nedaplatin; biND, bilateral neck dissection; uniND, unilateral neck dissection; RF, radial forearm free flap; RAMC, rectus abdominis myocutaneous free flap; SM, segmental mandibulectomy. ^*∗*^Time interval between the end of RT and the day of surgical debridement and fibula flap reconstruction. ^*∗∗*^Ipsilateral: ORN occurred at the same side of primary tumor exposed to radiation. Contralateral: ORN occurred at contralateral side of primary tumor exposed to radiation. ^*∗∗∗*^Extent of segmental mandibulectomy was classified according to the CAT classification, described in detail in the text.

**Table 2:** Histopathological results of bone specimens.

Case	Anterior margin			Medial area	Posterior margin		
				(Central area of bone destruction)			
	Cortical bone^*∗*^		Cancellous bone	Cancellous bone	Cortical bone^*∗*^		Cancellous bone
	Inferior border	Middle level			Inferior border	Middle level	
1	*NNNN*	*VVVV*	*VVVV*	*VVVV*	*VVVV*	*VVVV*	*VVVV*
2	*NNNN*	*NNNN*	*VVVV*	*VVVV*	*NNNN*	*NVNN*	*VVVV*
3	NVNV	*VVVV*	*VVVV*	VNVV	*VVVV*	*VVVV*	*VVVV*
4	*NNNN*	VNNN	*VVVV*	*NNNN*	*NNNN*	*NNNN*	NVNN
5	*NNNN*	*NNNN*	*VVVV*	NVNV	NVNN	*VVVV*	*VVVV*
6	*NNNN*	NVNN	*VVVV*	*VVVV*	*NNNN*	*NNNN*	*VVVV*
7	*VVVV*	*VVVV*	*VVVV*	*VVVV*	*VVVV*	*VVVV*	*VVVV*
8	*NNNN*	*NNNN*	*VVVV*	*VVVV*	NVNN	*NNNN*	*NNNN*
9	*NNNN*	*NNNN*	*VVVV*	*VVVV*	NVNN	*NNNN*	*VVVN*
10	*NNNN*	*VVVV*	*VVVV*	*VVVV*	*VVVV*	*VVVV*	*VVVV*
11	*NNNN*	*NNNN*	*VVVV*	VVNN	*NNNN*	*NNNN*	VVNN

Concordance rate (%)^*∗∗*^	91	82	100	73	73	100	82

^*∗*^Cortical bone was histopathologically analyzed at two levels (near the inferior border and the middle level of the mandible).   ^*∗∗*^Independently evaluated by four observers. V, viable; N, necrotic.
